# Home monitoring by pulse oximetry of primary care patients with COVID-19: a pilot randomised controlled trial

**DOI:** 10.3399/BJGP.2022.0224

**Published:** 2023-02

**Authors:** Karin Smit, Roderick P Venekamp, Loeke A Krol, Geert-Jan Geersing, Lisette Schoonhoven, Karin AH Kaasjager, Frans H Rutten, Dorien LM Zwart

**Affiliations:** Department of General Practice, Julius Center for Health Sciences and Primary Care, University Medical Center Utrecht, Utrecht University, Utrecht.; Department of General Practice, Julius Center for Health Sciences and Primary Care, University Medical Center Utrecht, Utrecht University, Utrecht.; Department of General Practice, Julius Center for Health Sciences and Primary Care, University Medical Center Utrecht, Utrecht University, Utrecht.; Department of General Practice, Julius Center for Health Sciences and Primary Care, University Medical Center Utrecht, Utrecht University, Utrecht.; Department of Public Health, Healthcare Innovation and Evaluation and Medical Humanities, Julius Center for Health Sciences and Primary Care, University Medical Center Utrecht, Utrecht University, Utrecht.; Department of Acute Internal Medicine, Julius Center for Health Sciences and Primary Care, University Medical Center Utrecht, Utrecht University, Utrecht.; Department of General Practice, Julius Center for Health Sciences and Primary Care, University Medical Center Utrecht, Utrecht University, Utrecht.; Department of General Practice, Julius Center for Health Sciences and Primary Care, University Medical Center Utrecht, Utrecht University, Utrecht.

**Keywords:** COVID-19, pulse oximetry, general practice, oximetry, SARS-CoV-2

## Abstract

**Background:**

Pulse oximetry as a home or remote monitoring tool accelerated during the pandemic for patients with COVID-19, but evidence on its use is lacking.

**Aim:**

To assess the feasibility of home monitoring by pulse oximetry of patients aged ≥40 years with cardiovascular comorbidity and moderate-to-severe COVID-19.

**Design and setting:**

A primary care-based, open, pilot randomised controlled trial, with nested process evaluation, was undertaken in the Netherlands.

**Method:**

From November 2020 to June 2021, eligible patients presenting to one of 14 participating Dutch general practices were randomly allocated to regular measurement of peripheral oxygen saturation (at least three SpO_2_ measurements per day for 14 days) with a validated pulse oximeter or usual care.

**Results:**

All 41 participants (21 intervention, 20 usual care) completed the 45-day follow-up period. Overall, the intervention group performed 97.6% of protocolised measurements; the median daily measurement per participant was 2.7 (interquartile range 1–4). Hypoxemia (SpO_2_ <94%) was reported in 10 participants (in 52 measurements); of those, six consulted the GP as instructed. Participants reported a high feeling of safety (0–100 visual analogue scale): 71.8 for the intervention group versus 59.8 for the control (*P* = 0.09). Primary care consultations were similar across groups: 50 for the intervention versus 51 for the control. Eleven visits by 10 participants were made to the emergency department (eight from the intervention group versus three from usual care), of which six participants were hospitalised (five intervention versus one usual care). No participants were admitted to the intensive care unit or died during follow-up.

**Conclusion:**

Home monitoring of patients with moderate-to-severe COVID-19 by pulse oximetry appeared feasible; adherence was high, patients reported a high feeling of safety, while the number of primary care consultations remained similar to usual care.

## INTRODUCTION

A pulse oximeter is a small, easy-to-operate, non-invasive tool to measure the peripheral oxygen saturation (SpO_2_). Its use accelerated as a home or remote monitoring tool during the pandemic for patients with COVID-19. Indeed, in COVID-19, hypoxemia is a marked phenomenon in the disease trajectory of clinical deterioration mandating intensified treatment. Yet, patients may have hypoxemia without clinical perceptible symptoms (‘happy hypoxemia’). Given the key biological role of oxygen saturation and the detrimental effects of hypoxemia, regular SpO_2_ measurements seem to hold promise, in particular for patients with COVID-19 who are at risk of complications such as those with cardiovascular comorbidity.^1^^–^5 Timely detection of hypoxemia could facilitate prompt referral for intensified treatment and thereby improve prognosis.^6^^–^9 However, studies on its feasibility, effectiveness, safety, and patients’ perceptions are scarce, especially in at-risk patients.^10^^,^^11^ A recent large trial among patients with suspected or confirmed COVID-19 found no difference in the number of days alive and out of hospital between patients who received home monitoring with pulse oximetry and home monitoring without pulse oximetry.^12^ However, this trial predominantly included patients with mild symptoms, with only 84 of 1217 participants with COVID-19 being hospitalised during follow-up. It is particularly important to study the use of pulse oximetry in primary care patients with COVID-19 who are at risk of complications, as no intervention comes without potential side effects; for home monitoring of SpO_2_, that is, the use of the pulse oximeter itself or the behaviour of the end-user. Regarding the pulse oximeter itself, most pulse oximeters used in the open population are consumables with a regulatory CE mark but without approval for medical use by the Food and Drug Administration (FDA) or the International Organization for Standardization (ISO) standards. The FDA and ISO require an adequate test against direct arterial oxygen saturation measurements in the range 70%–100% with <3% difference.^13^^,^^14^ The widely used consumables fall short for the detection of (severe) hypoxemia.^15^ This may lead to a false sense of security in both patients and physicians and, importantly, may leave clinical deterioration unnoticed. On the other hand, one could argue that regular checks of SpO_2_ levels may induce anxiety and consequently result in overuse of healthcare facilities by patients.

Therefore, a primary care-based, open, pilot randomised controlled trial (RCT) was conducted. The aim was to assess the feasibility of a trial of home monitoring by pulse oximetry for patients aged ≥40 years with cardiovascular comorbidity and moderate-to-severe COVID-19 compared with usual care.

**Table:** How this fits in

During the pandemic, home or remote monitoring of patients with COVID-19 by pulse oximetry took off. However, studies on its use are scarce. This pilot randomised controlled trial showed that home monitoring of patients with moderate-to-severe COVID-19 with a validated pulse oximeter is feasible; adherence was high, patients reported a high feeling of safety, while the number of primary care consultations remained similar to usual care. These pragmatic findings form an important building block for safe implementation of pulse oximetry as a home monitoring tool in primary care.

## METHOD

### Trial design

Between November 2020 and June 2021, an open-label, individually randomised (one-to-one) controlled pilot trial, with nested process evaluation, was conducted in Dutch primary care.

### Public and patient involvement

Patients of the Patient and Family Advisory Council from the ZonMw institution in the Netherlands provided input in defining research questions, outcomes, and data collection at the design stage of the study. This group consisted of middle-aged patients with chronic or oncologic disease who received structured care via a transmural care programme, thus belonging to the COVID-19 risk group. The results will be shared with the patients involved.

### Participants

Patients were aged ≥40 years with cardiovascular comorbidity who presented to the GP with moderate-to-severe COVID-19 symptoms. Moderate-to-severe symptoms were defined as at least 3 days with a body temperature ≥37.5°C and either a) new onset of symptoms of respiratory tract infection; b) a feeling of shortness of breath; and/or c) sudden exhaustion. They were patients for whom it was considered necessary to closely follow-up, according to the GP. The following patients were excluded: those requiring hospital admission; those with known severe anaemia (pulse oximetry can be inaccurate and SpO_2_ overestimated in this situation); those with inadequate mastery of Dutch language; and those unwilling to sign informed consent or adhere to study procedures.^16^ A specific cut-off value for severe anaemia was not defined in the exclusion criteria. In practice, a patient with severe anaemia would need transfusion or hospital admission.

GPs from 14 participating general practices in the vicinity of Utrecht informed potentially eligible participants about the study verbally and via a patient information letter. Those who were interested, and tested positive for COVID-19, were asked for consent to share their contact details with the University Medical Center (UMC) Utrecht research team for eligibility screening. Eligible patients who expressed interest in trial participation were visited at home under safe circumstances with COVID-19 protection to obtain full-written informed consent. Next, the study physician accessed a trial randomisation website for concealed study treatment assignment via a computer-generated sequence list developed by an independent data manager, that is, home monitoring by pulse oximetry or usual care. The study physician informed the GP about the randomisation result.

### Intervention

All participants in the intervention group received an FDA-approved for medical use pulse oximeter (Nonin 3230); together with verbal, written, and visual instructions to measure their SpO_2_ levels at rest three times a day for 14 consecutive days.^14^^,^^17^^,^^18^ If SpO_2_ was <94%, participants were instructed to perform an additional measurement after 5 min of rest. In cases of persisting hypoxemia, participants were instructed to contact their GP.^19^^,^^20^ In cases where participants felt unwell or experienced worsening in clinical condition, they were also instructed to contact their GP, irrespective of SpO_2_ levels.

Before distribution, all pulse oximeters were registered, checked, and released by the Department of Medical Technology and Clinical Physics at the UMC Utrecht.^21^

### Data collection

At baseline, a short interviewer-administered questionnaire was completed, including demographic data and the 12-item WHODAS 2.0 (World Health Organization Disability Assessment Schedule 2.0), which is a generic assessment instrument developed by the WHO to measure health and disability (scale: 0 = no disability to 48 = high disability).^22^

Patients in the intervention group recorded their oxygen saturation in a paper diary three times a day for 14 days. After 14 days, participants reported their overall feeling of safety over the previous 2 weeks on a 0 (completely unsafe) to 100 (completely safe) visual analogue scale by phone.

At the end of the 45-day follow-up period, participants completed the 12-item WHODAS 2.0 again and those in the control group were asked by phone if they had used a pulse oximeter at home after study enrolment. Healthcare utilisation was captured by retrieving patients’ primary care electronic health record data.

### Outcomes

The primary outcome was feasibility of a trial of home monitoring by pulse oximetry defined as successful inclusion of approximately 50 participants within 6 months who were willing to a) be randomised; and b) adhere to study procedures.

Secondary outcomes included quantitative data about the use of pulse oximetry in practice (see process evaluation below), patient-reported feeling of safety over the first 2 weeks, and disability-free survival after 45 days as determined by percentage change in 12-item WHODAS 2.0 sum score from baseline to 45 days: number of GP consultations, number of emergency department (ED) visits, hospital and/or intensive care unit (ICU) admissions, number of days alive at home, and all-cause mortality.

### Process evaluation

In a process evaluation alongside the trial, the study examined how the intervention was used in practice in terms of fidelity (intervention carried out as planned), dose (intervention was used as long and frequently as planned), adjustments (whether made to the intervention and why), and reach (whether the intended audience had been reached).^23^ For this, data were used on healthcare utilisation in both groups, and data from the paper diary in the intervention group.

### Sample size considerations

A formal sample-size calculation was not performed for this feasibility pilot trial. It was initially aimed to randomise approximately 50 participants, a number deemed to be sufficient to assess the feasibility of the trial.

### Statistical analysis

All analyses were performed according to the intention-to-treat principle. Baseline characteristics were presented descriptively. For between-group comparisons, the study used crude analysis with χ^2^ test or Fisher’s exact test, Mann-Whitney *U*, or independent samples *t*-test, where appropriate. A two-tailed *P*-value of <0.05 was considered statistically significant. All data were analysed using IBM SPSS Statistics (version 26.0).

## RESULTS

**Figure 1.**
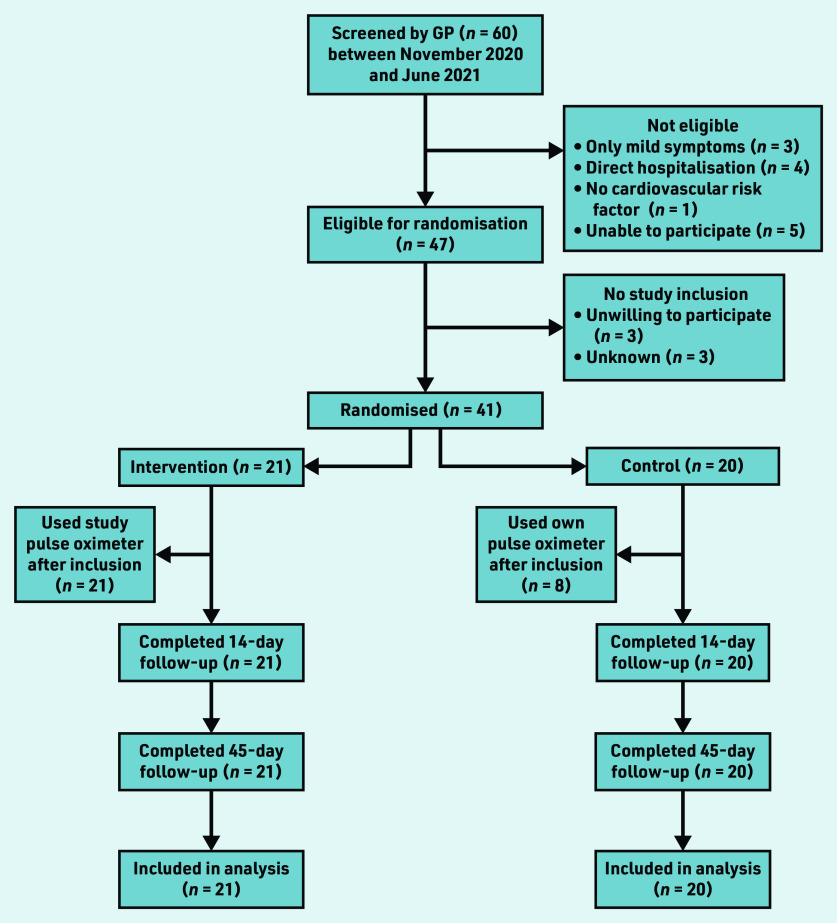
*Flowchart of study participants.*

Between November 2020 and June 2021, 60 patients were screened for participation by the GP; of those, 41 patients were eligible and randomised. Twenty-one were assigned to the intervention and 20 to the control group (Figure 1). All participants were tested positive for SARS-CoV-2 with polymerase chain reaction (PCR) before inclusion.

Follow-up data were fully captured, except for the 45-day WHODAS 2.0 questionnaire, which was not completed by one participant in the intervention arm.

Participants’ mean age was 64.2 years (standard deviation [SD] 10.8 years), and 56.1% (*n* = 23) were male (61.9% [*n* = 13] in the intervention group and 50.0% [*n* = 10] in the control group). Hypertension was the most common cardiovascular comorbidity (68.3%; *n* = 28) followed by hypercholesterolemia (56.1%; *n* = 23). Except for sex, baseline characteristics did not substantially differ across groups (Table 1).

**Table 1.** Baseline characteristics for the intervention and usual care group participants

**Characteristic**	**Total (*n*= 41)**	**Intervention (*n*= 21)**	**Usual care (*n*= 20)**
**Mean age, years (SD)**	64.2 (10.8)	63.2 (10.0)	65.3 (11.7)
**Male sex,** ***n* (%)**	23 (56.1)	13 (61.9)	10 (50.0)
**Mean BMI, mg/kg^2^ (SD)** ^a^	28.4 (4.5)	28.7 (4.5)	28.0 (4.7)
Obesity, BMI ≥30 mg/kg^2^, *n* (%)	9 (22.0)	5 (23.8)	4 (20.0)
**Hypertension,** ***n* (%)**	28 (68.3)	13 (61.9)	15 (75.0)
**Smoking status,** ***n* (%)**			
Never	12 (29.3)	4 (19.0)	8 (40.0)
Current or prior	11 (26.8)	6 (28.6)	5 (25.0)
Unknown	18 (43.9)	11 (52.4)	7 (35.0)
**Diabetes,** ***n* (%)**	11 (26.8)	6 (28.6)	5 (25.0)
**Hypercholesterolaemia,** ***n* (%)**	23 (56.1)	13 (61.9)	10 (50.0)
**Coronary artery disease,** ***n* (%)^b^**	6 (14.6)	2 (9.5)	4 (20.0)
**Chronic kidney disease (eGFR <60 ml/min),** ***n* (%)**	8 (19.5)	5 (23.8)	3 (15.0)
**Ischaemic or haemorrhagic stroke,** ***n* (%)**	5 (12.2)	4 (19.0)	1 (5.0)
**Chronic obstructive pulmonary disease,** ***n* (%)^c^**	14 (34.1)	6 (28.6)	8 (40.0)
**Immunocompromised,** ***n* (%)**	3 (7.3)	1 (4.8)	2 (10.0)
**Charlson Comorbidity Index,** **modified, 8/17 items (SD)^d^**	1.20 (1.14)	1.24 (1.22)	1.15 (1.09)
**COVID-19 confirmed with PCR** **SARS-CoV-2-test,** ***n* (%)**	40 (97.6)	21 (100)	19 (95.0)
**Median number of days with symptoms before** **inclusion (range)**	7.0 (0–20)	7.0 (0–15)	6.5 (2–20)

a *Missing* n*= 12.*

b *Including angina pectoris, myocardial infarction, and heart failure.*

c *Including asthma and chronic obstructive pulmonary disease.*

d *Data of 8/17 items of the International Classification of Diseases, 10^th^ Revision version of the Charlson Comorbidity Index was used. Total score range 0–10. BMI = body mass index. eGFR = estimated glomerular filtration rate. PCR = polymerase chain reaction. SD = standard deviation.*

### Use of the intervention

All participants from the intervention group used the pulse oximeter and a total of 727 SpO_2_ readings were reported; median daily measurements per patient was 2.7 (interquartile range 1–4). Overall, the intervention group performed 97.6% of protocolised measurements (adherence to measurements) (data available from the corresponding author on request).

**Figure 2.**
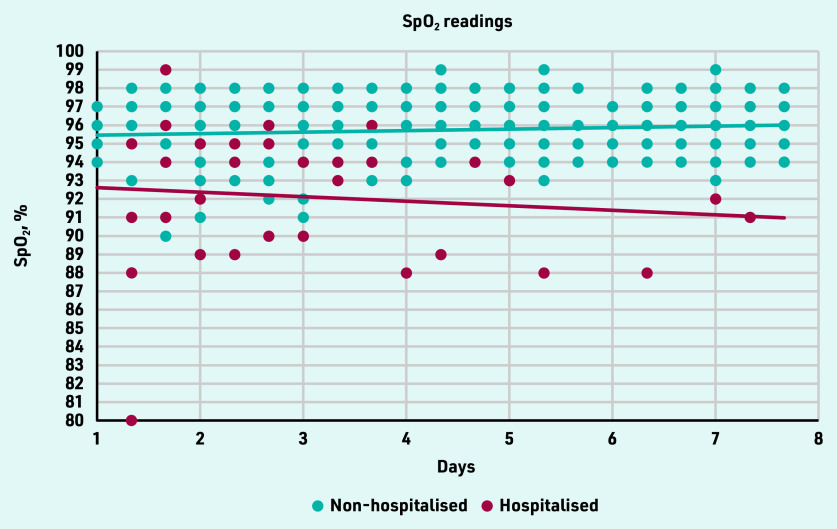
*SpO_2_ readings for the first 7 days of the 21 intervention group participants. Readings of participants who needed hospital admission are shown until hospitalisation.*

Hypoxemia (SpO_2_ <94%) was measured 52 times in 10 participants. Of these, six contacted their GP as instructed (adherence to contacting the GP: 60.0%). Figure 2 gives an overview of SpO_2_ readings of the intervention group in the first 7 days. The readings of participants who needed hospital admission are presented until hospitalisation.

No adjustments to the study protocol were necessary during the study.

In the usual care group, eight participants (40.0%) reported to have used a pulse oximeter in the 14 days following randomisation (Figure 1).

### Feeling of safety

After 14 days, participants reported a high feeling of safety: 71.8 (SD 19.1, range 30–100) in the intervention group versus 59.8 (SD 24.5, range 10–100) in the control group (*P* = 0.09). When including only the 35 non-hospitalised participants in the analysis, the feeling of safety was 73.8 (SD 17.8) versus 57.6 (SD 23.3), respectively (*P* = 0.03). When including only the 10 participants who attended the ED and/or were hospitalised (*n* = 6) in the analysis, the feeling of safety in the intervention group was 68.3 (SD 20.7) versus 63.3 (SD 32.1) in the control group (*P* = 0.85) (data available from the corresponding author on request).

### Disability score (WHODAS)

After 45 days, participants reported a decrease in disability as measured with WHODAS 2.0 compared with baseline. The intervention versus control was 53.2% versus 65.7% (*P* = 0.42). When including only the 35 non-hospitalised participants in the analysis, the decrease was 53.2% versus 64.5%, respectively (*P* = 0.52). Scores on WHODAS 2.0 questionnaire are presented in Table 2.

**Table 2.** Disability score WHODAS 2.0 for the intervention and usual care group participants

**Category**	**Total (*n*= 41)**	**Intervention (*n*= 21)**	**Usual care (*n*= 20)**	***P*-value**
**Total 12-item WHODAS 2.0 score at baseline, mean (SD)^a^**	19.2 (10.9)	19.7 (11.6)	18.8 (10.4)	0.73
**Total 12-item WHODAS 2.0 score after 45 days, mean (SD)^a^**	6.1 (6.9)	8.4 (8.4)	3.9 (4.1)	0.10
**Percentage decrease after 45 days (SD)**	59.4 (48.1)	53.2 (39.4)	65.7 (55.7)	0.42
**Percentage decrease after 45 days in non-hospitalised participants (SD)**	59.6 (50.2)	53.2 (41.0)	64.5 (57.0)	0.52

a *Including rating 12 items on a five-point scale. Total score is computed by summarising scores. Zero represents no difficulties, 48 represents most severe difficulties. SD = standard deviation. WHODAS = World Health Organization Disability Assessment Score.*

### Healthcare utilisation and health outcomes

Healthcare resource use and health outcomes during the 45-day follow-up period are presented in Table 3.

**Table 3.** Healthcare utilisation and other health-related secondary outcomes for the intervention and usual care group participants

**Category**	**Total (*n*= 41)**	**Intervention (*n*= 21)**	**Usual care (*n*= 20)**	***P*-value**
**GP contacts**				
**Number of COVID-19 related GP contacts, *n***	101	50	51	0.550
Because of low oxygen saturation measurement,*n* (%)	15 (14.8)	12 (24.0)	3 (5.9)	0.213
**Patients with at least one GP contact after inclusion, *n* (%)**	31 (75.6)	15 (71.4)	16 (80)	0.523
**Median GP contacts per patient during 45 days (range)**	3.0 (1–12)	2.0 (1–12)	3.0 (1–8)	0.550
**Hospital visits and admission**				
**Total number of ED visits, *n* (%)**	11 (26.8)	8 (38.1)	3 (15.0)	0.513
**Patients admitted to hospital, *n* (%)**	6 (14.6)	5 (23.8)	1 (5.0)	0.184
Median length of stay, days (range)	5.0 (3–16)	3.0 (3–16)	7.0 (0.0)	n/a
Non-invasive oxygen treatment,*n* (%)	6 (100)	5 (100)	1 (100)	0.180
**ICU admissions, *n* (%)**	0 (0.0)	0 (0.0)	0 (0.0)	n/a

**Secondary diagnosis**				
Bacterial superinfection, *n* (%)	10 (24.4)	6 (28.6)	4 (20.0)	0.720
Pulmonary embolism,*n* (%)	1 (2.4)	1 (4.8)	0 (0.0)	n/a
**Treatment with dexamethasone, *n* (%)**	7 (17.1)	5 (23.8)	2 (10.0)	0.410

**Days alive at home, mean (SD)**	43.5 (6.1)	42.4 (8.3)	44.7 (1.6)	0.239

**Deaths, *n* (%)**	0 (0.0)	0 (0.0)	0 (0.0)	n/a

*ED = emergency department. ICU = intensive care unit. n/a = not applicable. SD = standard deviation.*

In total, 31 participants had at least one contact with their GP after inclusion (intervention 71.4% [*n* = 15] versus control 80.0% [*n* = 16], *P* = 0.52). The number of primary care consultations was similar across groups: intervention 50 versus control 51. Median time to first contact was 3.0 days (intervention 1.0 day versus control 4.8 days, *P* = 0.07) (data available from the corresponding author on request).

During follow-up, 10 patients visited the ED 11 times: eight from intervention versus three usual care (*P* = 0.51). This led to hospitalisation of six participants (intervention *n* = 5 versus control = 1; *P* = 0.18) with a median length of stay of 5.0 days (intervention 3.0 days versus control 7.0 days [*n* = 1]). No participants were admitted to the ICU (Table 3).

There was no significant difference in number of days alive at home between groups: intervention 42.4 days (SD 8.3) versus control 44.7 days (SD 1.6) (*P* = 0.24). No participants died during the study (Table 3).

## DISCUSSION

### Summary

This pilot RCT showed that (a trial of) home monitoring of patients with cardiovascular comorbidity and moderate-to-severe COVID-19 with a validated pulse oximeter is feasible; patients were willing to participate, there was a high level of adherence to pulse oximetry measurements, and no protocol changes were necessary. Patients reported a high feeling of safety, which tended to be higher in those using a pulse oximeter, and using the tool did not lead to an increase in primary care consultations compared with usual care.

The hospitalisation rate in the intervention group was higher than in the control group, and the median length of stay in hospital was shorter in the intervention group than in the control group. These differences must be interpreted with caution because it may be a chance finding given the small numbers. It could, however, be owing to detection of ‘silent’ hypoxemia in the intervention arm, which was followed by adequate referral to hospital.

### Strengths and limitations

The authors performed, to their knowledge, the first entirely primary care-based pilot RCT to assess the feasibility of a trial of home monitoring by pulse oximetry of high-risk patients with COVID-19. Most of the eligible patients were willing to be randomised and adhere to study procedures. The lower than anticipated participation rate was partly owing to a decline in SARS-CoV-2 prevalence in the Netherlands at the end of the study period, and a more widespread use of pulse oximetry by patients with COVID-19. In the study, 8/20 (40.0%) control participants used an ‘own’ pulse oximeter at least once during the study. This could have reduced the contrast between groups, meaning a possible reduction of effect.

Overall, in the pilot trial, the use of pulse oximetry tended to reduce anxiety and there were no unsafe situations. A limitation is that an electronic real-time connection was not used between pulse oximeter and medical assistance, so it is possible that detection of hypoxemia measurements was missed or delayed, even though explicit instructions were given to patients when to contact the GP.

### Comparison with existing literature

In line with the present study’s findings, adherence to pulse oximetry use was high in previous observational studies among patients with COVID-19.^24^^,^^25^ Adherence was, however, much lower in a recent US-based RCT among 2097 patients with suspected or confirmed COVID-19 in the community in which a lenient protocol, as part of routine care, was applied; where only 77% of participants in the intervention group performed SpO_2_ measurement at least once during the study period.^12^ The authors found no differences in hospitalisation rates and mortality between confirmed patients with COVID-19 in the intervention and control groups. The present study found the number of ED visits (26.8%; *n* = 11) and number of hospital admissions (14.6%; *n* = 6) were more comparable with those observed in a 2020 prospective cohort study.^26^ Yet, the present study’s results were slightly higher, which is possible because the study included a specific population with cardiovascular comorbidity. The results could not be compared with a 2022 systematic review of pulse oximetry as a remote patient monitoring tool, because the study could not identify clear evidence for the effect on health outcomes.^11^

While it has been suggested by expert opinion that pulse oximetry could induce anxiety, the study found the opposite. Patients reported they felt safe when using the pulse oximeter, which is comparable with a high feeling of safety reported by patients in a 2020 case-control study.^27^

In 45 days, the mean percentage decrease in disability score, as measured with WHODAS 2.0, was 53.2% in the intervention group and 65.7% in the control group. Currently, as far as the authors are aware, there are no known studies that define which change would be deemed a clinically relevant improvement. The large decrease from baseline observed in the present study is likely explained by the serious clinical impact of COVID-19, where participants at baseline scored a high score above the 95th percentile compared with the general population. This decreased to a disability score around the 75th percentile (summary score 6.5) at day 45, which indicates that patients with COVID-19 have residual disabilities after their infection.^22^

### Implications for practice

Home monitoring by pulse oximetry is already recommended by the WHO as part of a COVID-care package, and incorporated in UK guidelines for breathless, unwell, or high-risk patients with COVID-19.^28^ The study has shown that home monitoring with a validated pulse oximeter tended to increase the feeling of safety of participants compared with usual care (in which 40.0% used a pulse oximeter). To enhance patient safety, it is important that validated pulse oximeters are used as remote monitoring tools.

In conclusion, the pilot RCT showed that home monitoring of patients with moderate-to-severe COVID-19 with a validated pulse oximeter is feasible; adherence was high, patients reported a high feeling of safety, and the use of pulse oximetry did not result in an increase in primary care consultations compared with usual care. It is believed these findings are an important building block for safe implementation of pulse oximetry as a home monitoring tool in primary care.

## Data

Datasets are available. Any supplementary data can be requested from the corresponding author.
